# Supplementary material for: Organising the cell cycle in the absence of transcriptional control: Dynamic phosphorylation co-ordinates the Trypanosoma brucei cell cycle post-transcriptionally
Source: PLoS Pathog. 2019 Dec 12;15(12):e1008129. doi: 10.1371/journal.ppat.1008129 (PMC6907760; doi:10.1371/journal.ppat.1008129)
Supplement: S1 Methods — (DOCX) [file ppat.1008129.s001.docx]

Organising the cell cycle in the absence of transcriptional control: Dynamic phosphorylation co-ordinates the *Trypanosoma brucei* cell cycle post-transcriptionally

Corinna Benz,^1,2^ Michael D. Urbaniak^1^*

^1^ Biomedical and Life Sciences, Lancaster University, Bailrigg, Lancaster, LA1 4WG, UK

^2^ Present address: Institute of Parasitology, Biology Center, Czech Academy of Sciences, 37005 České Budějovice, Czech Republic.

* Corresponding author. Email [m.urbaniak@lancaster.ac.uk](mailto:m.urbaniak@lancaster.ac.uk)

**Supporting Information**

[**S1 Methods** 2](#_Toc21960359)

[**Cell culture and SILAC labelling** 2](#_Toc21960360)

[**Synchronisation by centrifugal counter flow elutriation** 2](#_Toc21960361)

[**Filter aided sample preparation (FASP)** 2](#_Toc21960362)

[**Separation of peptides from phosphopeptides by Fe-IMAC** 2](#_Toc21960363)

[**High pH reverse phase fractionation** 3](#_Toc21960364)

[**Selection of cell cycle regulated proteins and phosphorylation sites** 3](#_Toc21960365)

[**Peak time calculation** 4](#_Toc21960366)

[**Hierarchical clustering** 4](#_Toc21960367)

[**Cluster annotation enrichment analysis** 4](#_Toc21960368)

[**Data visualisation** 4](#_Toc21960369)

[**References** 4](#_Toc21960370)

# **S1 Methods**

## **Cell culture and SILAC labelling**

The Stable isotope labelling by amino acids in cell culture (SILAC) labelling of *T. brucei* 427 Lister procyclic form (Pcf) cells was performed as described previously [1, 2]. In brief, log phase parasites were passaged into SDM-79 SILAC media (SDM-79 medium lacking L-arginine and L-lysine) supplemented with 10% dialysed FCS (1000 MWCO, Dundee Cell Products) and the standard concentration of either normal isotopic abundance L-arginine and L-lysine (SDM-79, referred to as Light label) or ^13^C_6_ L-arginine and 4,4,5,5-D_4_ L-lysine (SDM-79 + R6K4, referred to as Medium label) or with ^13^C_6_,^15^N_4_L-arginine and ^13^C_6_,^15^N_2_ L-lysine (SDM-79 + R10K8, referred to as Heavy label). The stable isotope-labelled amino acids were obtained from CK Isotopes, UK. Cells were allowed to grow in the respective medium for more than 7 cell divisions before cultures were enlarged to a volume of 200-300 ml at a final concentration of 1-2 × 10^7^ cells/ml for the elutriated samples (light and heavy label). To produce the medium-SILAC standard for use as an asynchronous control, a single batch of 1L of medium-labelled cells was grown to a density of 1 × 10^7^ cells/ml to minimise variability.

## **Synchronisation by centrifugal counter flow elutriation**

Elutriation was performed essentially as described in [3]. Briefly, logarithmic phase Pcf cells (5 × 10^6^ - 1.5 × 10^7^/ml) were harvested by centrifugation at 1,000 × g for 10 min at room temperature (RT) and resuspended in 20 ml Pcf elutriation buffer (PBS + 25% SDM-79). A maximum number of 3 × 10^9^ Pcf were used for each elutriation. For early and late G1 samples, 150 ml fractions eluting at 18 and 20 ml/min (early) and 22 and 24 ml/min (late) were harvested by centrifugation and processed for proteomic analysis as described below. For some late G2/M samples, the pump speed was gradually increased and fractions of about 100 ml collected and discarded to deplete the sample of G1 and S phase cells. The remaining cells (highly enriched in G2/M) were eventually collected at a pump speed of 35 ml/min. For any other samples, 150 ml fractions eluting at 18, 20 and 22 ml/min were harvested by centrifugation and resuspended in the appropriate amount of SDM-79 for further cultivation. Samples for proteomics and flow cytometry were withdrawn at appropriate time points.

To follow up on specific proteins in cell lines bearing epitope-tagged versions of the respective proteins, elutriation was performed as described above except with slightly smaller culture sizes (100-150ml). For SmOxP9-based cell lines [4], 100 ml fractions were collected and the fraction eluting at 18 ml/min was harvested by centrifugation and resuspended in the appropriate amount of SDM-79 for further cultivation. Flow cytometry samples, cell lysates for western blots and microscopy slides were prepared at different time points following elutriation as specified in the respective figures.

## **Filter aided sample preparation (FASP)**

Synchronised samples from two time points (light- and heavy-labelled) and one asynchronous control sample (medium-labelled) were thawed and mixed (2 × 10^8^ cells per sample, giving a total of 6 × 10^8^ cells). The proteins were solubilized with SDS and tryptic peptides generated by an adaptation of the filter aided sample preparation procedure as described previously [2, 5], except using a 1:50 w/w trypsin to protein ration of Trypsin Gold (Promega). The digested peptide solution was then removed from the column, diluted to 3 ml with ABC and acidified with 0.1% TFA before desalting using a 500 mg C_18_ column (SepPak, Waters), lyophilisation and storage at -80 ^0^C.

## **Separation of peptides from phosphopeptides by Fe-IMAC**

Separation of peptides from phosphopeptides using Fe-IMAC was performed as described by Ruprecht *et al* [6] with minor modifications. An analytical Fe-IMAC column (4 × 50 mm ProPac IMAC-10, Thermo Fisher Scientific) connected to an LC Packing Famos HPLC was charged with FeCl_3_ as described in (Ruprecht et al. 2015). Buffer A (30% MeCN, 0.07% TFA) was then used to wash and equilibrate the column before a blank run was performed. The gradient consisted of 0-3 min 100% buffer A at 50 µl/min, 3-4 min 100% buffer A at 50-150 µl/min, 4-15 min 100% buffer A at 150 µl/min, 15-16 min 100% buffer A at 150-100 µl/min, 16-76min 0-45% buffer B (30% MeCN, 0.5% NH_4_OH) at 100 µl/min, 76-77 min 100% buffer B at 100 µl/min, 77-82 min 100% buffer B at 100-200 µl/min, 82-85 min 100-0% buffer B at 200µl/min and 85-115min 100% buffer A at 200 µl/min. Lyophilised tryptic digests were resuspended in 100 µl of buffer A before injection onto the column. Fractions were collected every 2 min in low protein binding micro centrifuge tubes (Eppendorf Protein lo-bind), with elution of peptides monitored by absorbance at 280 nm. Early eluting fractions (<6 min) corresponding to peptides and later eluting fractions corresponding to phosphopeptides (~30 min) were lyophilised and stored at -80 ^0^C prior to further processing.

## **High pH reverse phase fractionation**

Samples were fractionated using the High pH Fractionation kit (ThermoFisher) according to the manufacturer’s instructions with the following modifications. Lyophilised (phospho)peptides were resuspended in 100µl 5 mM NH_4_OH and applied to the pre-treated columns (MeCN, 0.1 % TFA, and two 5 mM NH_4_OH washes). The flow-through was reapplied before being collected and desalted using C_18_ microspin columns (Harvard Apparatus). The columns were then washed 3 times with 5 mM NH_4_OH and the (phospho)peptides eluted with different concentrations of MeCN (2, 3, 4, 6, 10 and 50% MeCN). The eluates were concatenated into five fractions as follows: F1 = 2% + 50% eluates, F2 = 3% eluate, F3 = 4% eluate, F4 = 6% eluate, F5 = 10% eluate + desalted flow through. The concatenated fractions were then lyophilised prior to analysis by LC-MS/MS.

## **Selection of cell cycle regulated proteins and phosphorylation sites**

Prior to further analysis, the outputs from MaxQuant [7] were filtered to remove known contaminants and reverse sequences, and any SILAC ratios with greater ≥100% variation or phosphorylation sites with a localisation probability < 0.95 were excluded. The 18 biological replicates were grouped into six cell cycle time points (early G1, EG1; late G1, LG1; early S, ES; late S, LS, early G2/M, EG2M; late G2M, LG2M) on the basis of the proportion of cells in G1, S and G2/M phases determined by flow cytometry analysis of propidium iodide stained cells (S1 Table). The SILAC ratios of the individual time points were averaged, and the data set filtered to remove any protein groups or phosphorylation sites that were not observed at all six cell cycle time points. The averaged ratios obtained compare an individual cell cycle time point (i.e. EG1, LS) to the same medium labelled asynchronous population (M_Asyn_), which therefore constitutes an invariable internal standard that can be used to directly compare different cell cycle time points. We used the averaged EG1 sample (95-97% G1), which represents the start of the cell cycle, to normalize the data to give ratios relative to the abundance at the start of the cell cycle.

$${Log}_{2} \frac{LS}{EG1}= {Log}_{2} \frac{L_{LS}}{M_{Asyn}}- {Log}_{2} \frac{H_{EG1}}{M_{Asyn}}$$

The maximum fold change (FC_Max_) across the cell cycle was calculated as the difference between the maximum and minimum abundance relative to EG1. A histogram of Log_2_ FC_Max_ demonstrated a non-normal distribution of data, with a peak centred on the median value that was skewed toward higher values (S1 Fig). We consider any protein with an FC_Max_ ≥ 1.5-fold or phosphorylation site with FC_Max_ ≥ 3-fold to be cell cycle regulated (CCR), threshold values that represent approximately twice the median FC_Max_ (2 × Median protein FC_Max_ = 1.44; 2 × Median phosphorylation site FC_Max_ = 2.99).

To make our data accessible to the scientific community, we have submitted the results of our study to TriTrypDB [8], enabling researchers to access the data presented here.

## **Peak time calculation**

To determine the order in which the CCR protein/phosphorylation site abundances reaches a maximum, peak time t_peak_ was calculated according to the procedure of Olsen et al [9]. The ratios (averaged, normalised to EG1) *r*_1_ to *r*_6_ were scaled to the unit interval [0, 1] and the six time points *t*_1_ to *t*_6_ assigned values 1 to 6. Then for each protein the peak time t_peak_ was calculated using a weighted mean of the expression ratio of maximal expression (*r*_i_ = 1) at time point *t*_i_ with respect to the adjacent time points (*t*_i-1_ and *t*_i+1_). Since the time points represent a continuous cycle, if the maximal expression was at *t*_1_ it was preceded by *t*_0_ with expression *r*_6_ and if maximal expression was at *t*_6_ it was followed by *t*_7_ = 7 with expression *r*_1_.

$$t_{peak}= \left\{ \begin{aligned} \frac{t_{i-1} \times r_{i-1}+ t_{i}r_{i}+ t_{i+1} \times r_{i+1}}{r_{i-1}+ r_{i}+ r_{i+1}}, \mathrm{if}\max r_{i}\mathrm{at} i \in[2,5] \\ \frac{t_{i}r_{i}+ t_{i+1} \times r_{i+1}+0 \times r_{6}}{r_{i}+ r_{i+1}+ r_{6}} , \mathrm{if}\max r_{i}\mathrm{at} i=1 \\ \frac{t_{i-1} \times r_{i-1}+ t_{i}r_{i}+ 7r_{i+1}}{r_{i-1}+ r_{i}+ r_{7}} , \mathrm{if}\max r_{i}\mathrm{at} i=6 \end{aligned} \right.$$

The profiles were then ordered in increasing t_peak_ to gain temporal map of the cell cycle rendered as a heat map of the unit interval scaled values, and projected on a polar plot of t_peak_ versus FC_Max_.

## **Hierarchical clustering**

To cluster the regulated protein/phosphorylation sites that show similar expression profiles, the ratios in each expression profile were Z-scored by subtracting the mean profile value and dividing by the standard deviation, and hierarchical clustering performed using Euclidean distance of the complete linkage after pre-processing with K-means using 1000 iteration and 10 restarts of 300 or 150 clusters for phosphorylation sites or proteins respectively [10]. The final clusters were defined using a minimum distance threshold of < 2.5, resulting in 30 phosphorylation site clusters and 29 protein abundance clusters.

## **Cluster annotation enrichment analysis**

Gene ontology (GO) term enrichment analysis of clusters was performed using the TriTrypDB curated GO Slim set contained within the integrated GO enrichment tool, using an uncorrected *P* value cut off of 0.05 [8]. Kinase motif enrichment analysis was conducted in Perseus [10] by searching the protein sequence flanking the identified phosphorylation site for linear motifs corresponding to potential kinase motifs, and using Fisher’s exact test to determine the correlation between the phosphorylation site clusters and identified kinase motifs using a threshold of a Benjamini-Hochberg false discovery rate < 0.05. In addition, previously identified PLK substrates and binders [11, 12] were mapped onto phosphorylation sites at the protein level, and enrichment examined using Fisher’s exact test as above.

## **Data visualisation**

Data were visualised in Perseus [10], with addition heat maps rendered in Morpheus (https://software.broadinstitute.org/morpheus) and proteins schematics generated in IBS [13].

# **References**

1. Urbaniak MD, Guther MLS, Ferguson MAJ. Comparative SILAC Proteomic Analysis of *Trypanosoma brucei* Bloodstream and Procyclic Lifecycle Stages. PloS one. 2012;7(5):e36619. doi: 10.1371/journal.pone.0036619.g001.

2. Urbaniak MD, Martin DM, Ferguson MA. Global quantitative SILAC phosphoproteomics reveals differential phosphorylation is widespread between the procyclic and bloodstream form lifecycle stages of *Trypanosoma brucei*. Journal of proteome research. 2013;12(5):2233-44. Epub 2013/03/15. doi: 10.1021/pr400086y. PubMed PMID: 23485197; PubMed Central PMCID: PMC3646404.

3. Benz C, Dondelinger F, McKean PG, Urbaniak MD. Cell cycle synchronisation of Trypanosoma brucei by centrifugal counter-flow elutriation reveals the timing of nuclear and kinetoplast DNA replication. Sci Rep. 2017;7(1):17599. Epub 2017/12/16. doi: 10.1038/s41598-017-17779-z. PubMed PMID: 29242601; PubMed Central PMCID: PMCPMC5730572.

4. Poon SK, Peacock L, Gibson W, Gull K, Kelly S. A modular and optimized single marker system for generating Trypanosoma brucei cell lines expressing T7 RNA polymerase and the tetracycline repressor. Open Biol. 2012;2(2):110037. Epub 2012/05/31. doi: 10.1098/rsob.110037. PubMed PMID: 22645659; PubMed Central PMCID: PMCPMC3352093.

5. Wisniewski JR, Zougman A, Nagaraj N, Mann M. Universal sample preparation method for proteome analysis. Nat Methods. 2009;6(5):359-62. doi: 10.1038/nmeth.1322. PubMed PMID: 19377485.

6. Ruprecht B, Koch H, Medard G, Mundt M, Kuster B, Lemeer S. Comprehensive and reproducible phosphopeptide enrichment using iron immobilized metal ion affinity chromatography (Fe-IMAC) columns. Mol Cell Proteomics. 2015;14(1):205-15. Epub 2014/11/15. doi: 10.1074/mcp.M114.043109. PubMed PMID: 25394399; PubMed Central PMCID: PMCPMC4288255.

7. Cox J, Mann M. MaxQuant enables high peptide identification rates, individualized p.p.b.-range mass accuracies and proteome-wide protein quantification. Nat Biotechnol. 2008;26(12):1367-72. doi: 10.1038/nbt.1511. PubMed PMID: 19029910.

8. Aslett M, Aurrecoechea C, Berriman M, Brestelli J, Brunk BP, Carrington M, et al. TriTrypDB: a functional genomic resource for the Trypanosomatidae. Nucleic Acids Res. 2010;38(Database issue):D457-62. doi: 10.1093/nar/gkp851. PubMed PMID: 19843604; PubMed Central PMCID: PMC2808979.

9. Olsen JV, Vermeulen M, Santamaria A, Kumar C, Miller ML, Jensen LJ, et al. Quantitative phosphoproteomics reveals widespread full phosphorylation site occupancy during mitosis. Sci Signal. 2010;3(104):ra3. doi: 10.1126/scisignal.2000475. PubMed PMID: 20068231.

10. Tyanova S, Temu T, Sinitcyn P, Carlson A, Hein MY, Geiger T, et al. The Perseus computational platform for comprehensive analysis of (prote)omics data. Nat Methods. 2016;13(9):731-40. Epub 2016/06/28. doi: 10.1038/nmeth.3901. PubMed PMID: 27348712.

11. McAllaster MR, Ikeda KN, Lozano-Nunez A, Anrather D, Unterwurzacher V, Gossenreiter T, et al. Proteomic identification of novel cytoskeletal proteins associated with TbPLK, an essential regulator of cell morphogenesis in Trypanosoma brucei. Mol Biol Cell. 2015;26(17):3013-29. Epub 2015/07/03. doi: 10.1091/mbc.E15-04-0219. PubMed PMID: 26133384; PubMed Central PMCID: PMCPMC4551316.

12. Hu H, Zhou Q, Li Z. A Novel Basal Body Protein That Is a Polo-like Kinase Substrate Is Required for Basal Body Segregation and Flagellum Adhesion in Trypanosoma brucei. J Biol Chem. 2015;290(41):25012-22. Epub 2015/08/15. doi: 10.1074/jbc.M115.674796. PubMed PMID: 26272611; PubMed Central PMCID: PMCPMC4599006.

13. Liu W, Xie Y, Ma J, Luo X, Nie P, Zuo Z, et al. IBS: an illustrator for the presenation and visualisation of biological sequences. Bioinformatics. 2015;31(20):3359-61.
