# Supplementary figures and images for: Organising the cell cycle in the absence of transcriptional control: Dynamic phosphorylation co-ordinates the Trypanosoma brucei cell cycle post-transcriptionally
Source: PLoS Pathog. 2019 Dec 12;15(12):e1008129. doi: 10.1371/journal.ppat.1008129 (PMC6907760; doi:10.1371/journal.ppat.1008129)

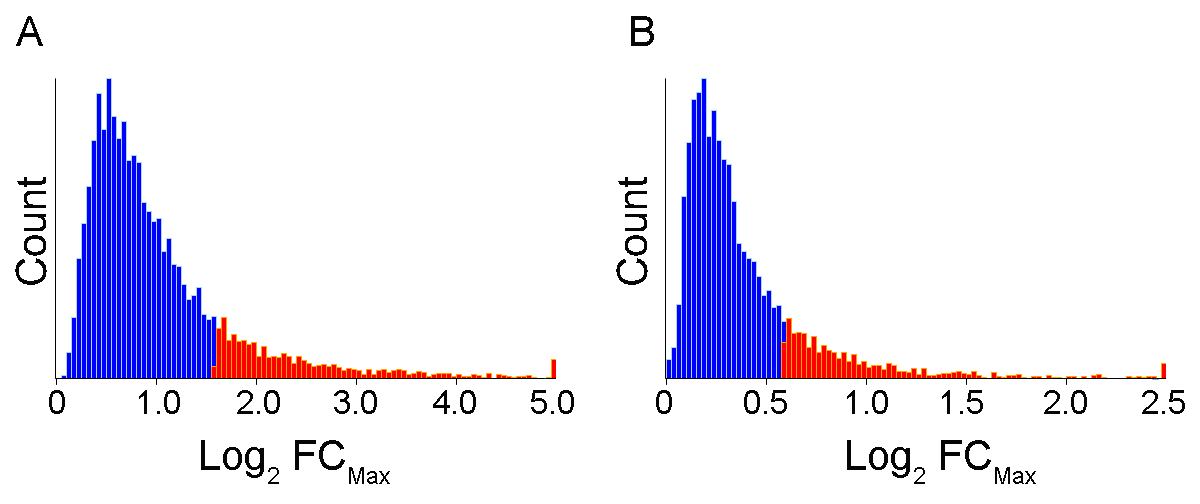

Supplement: S1 Fig — A. Phosphorylation site FCMax. B. Protein FCMax; Blue–Non–regulated, Red–Cell cycle regulated. Image prepared in Perseus [60]. (TIF) [file ppat.1008129.s002.tif]

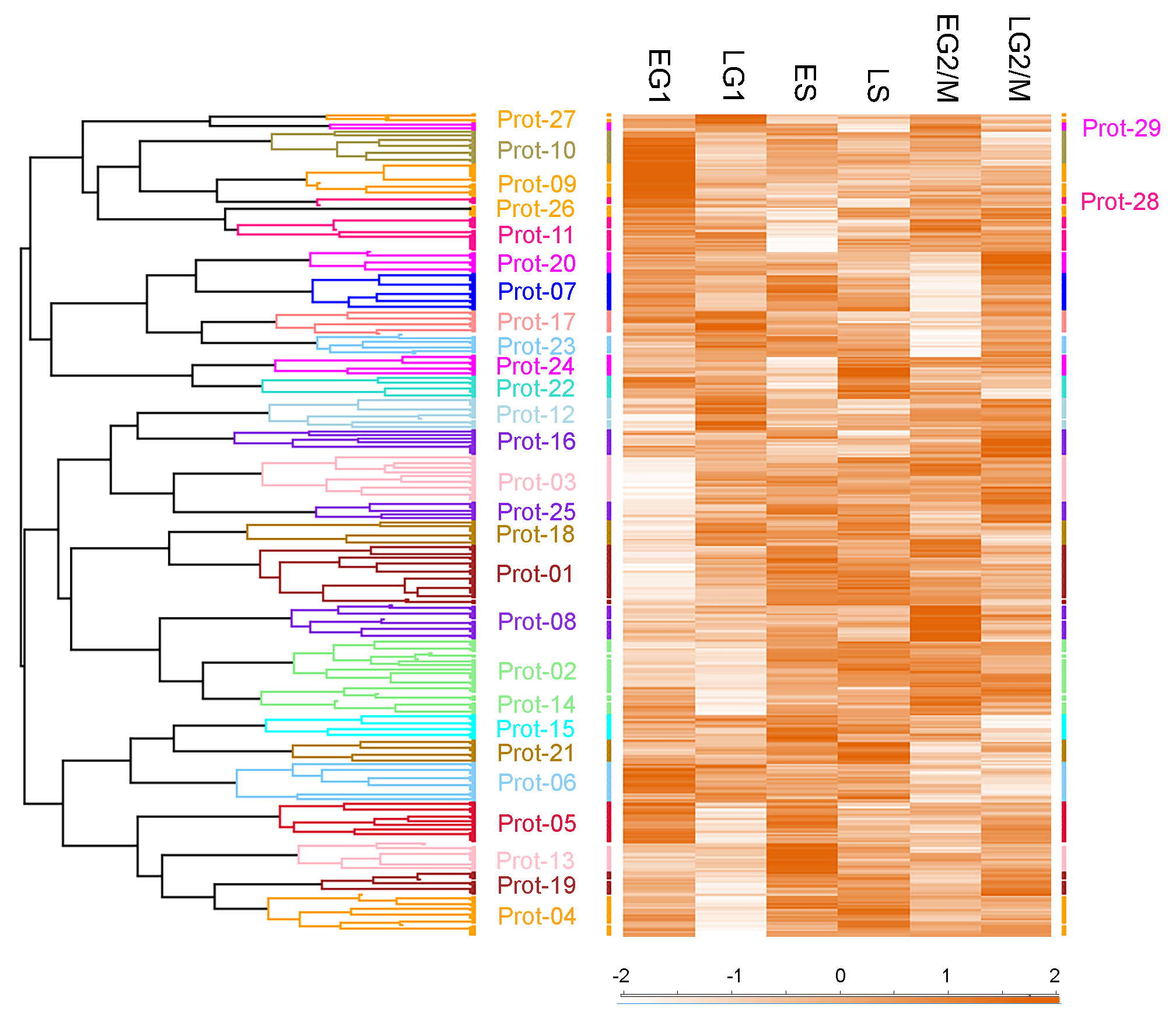

Supplement: S2 Fig — The protein ratios were Z-transformed and unrestrained hierarchical clustering performed using Euclidean distance of the complete linkage, with 29 clusters (Prot-01 to Prot-29) defined using a minimum distance threshold of < 2.5. The relationship between the clusters is rendered as a tree, and the Z-transformed phosphorylation site profiles are represented as a heat map. Image prepared in Perseus [60]. (TIF) [file ppat.1008129.s003.tif]

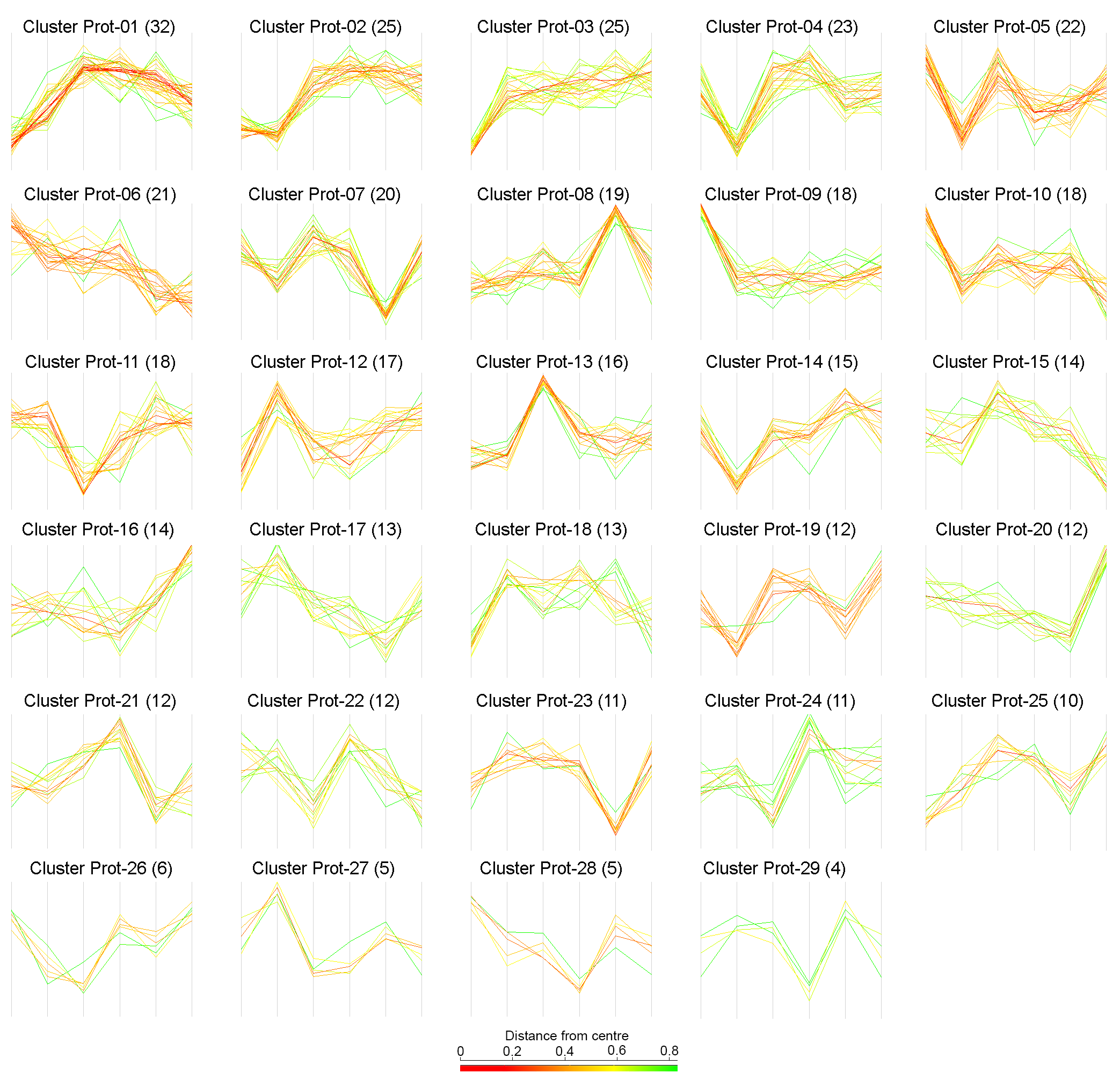

Supplement: S3 Fig — The protein ratios were Z-transformed and unrestrained hierarchical clustering performed using Euclidean distance of the complete linkage. The number of proteins in each cluster is given in brackets, and the profiles are coloured by the Euclidean distance from the centre (mean profile) of the cluster. Image prepared in Perseus [60]. (TIF) [file ppat.1008129.s004.tif]

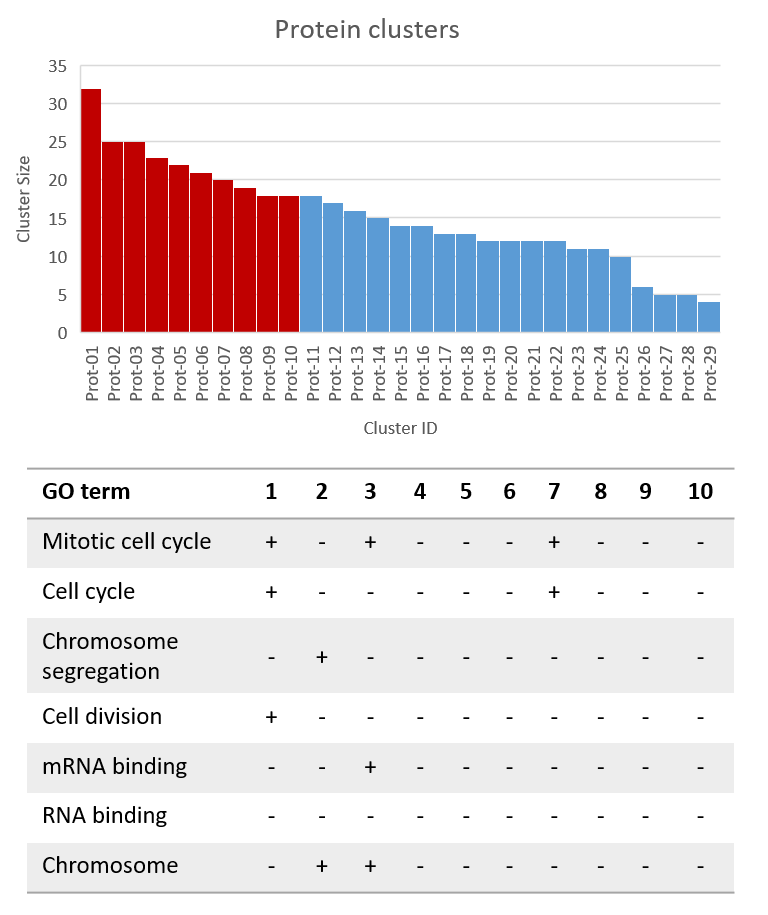

Supplement: S4 Fig — Gene Ontology (GO) enrichment analysis using GO Slim ontology with P < 0.05 was performed on the largest clusters representing >50% of the CCR proteins (red bars), and the occurrence of GO terms related to cell cycle tabulated. (TIF) [file ppat.1008129.s005.tif]

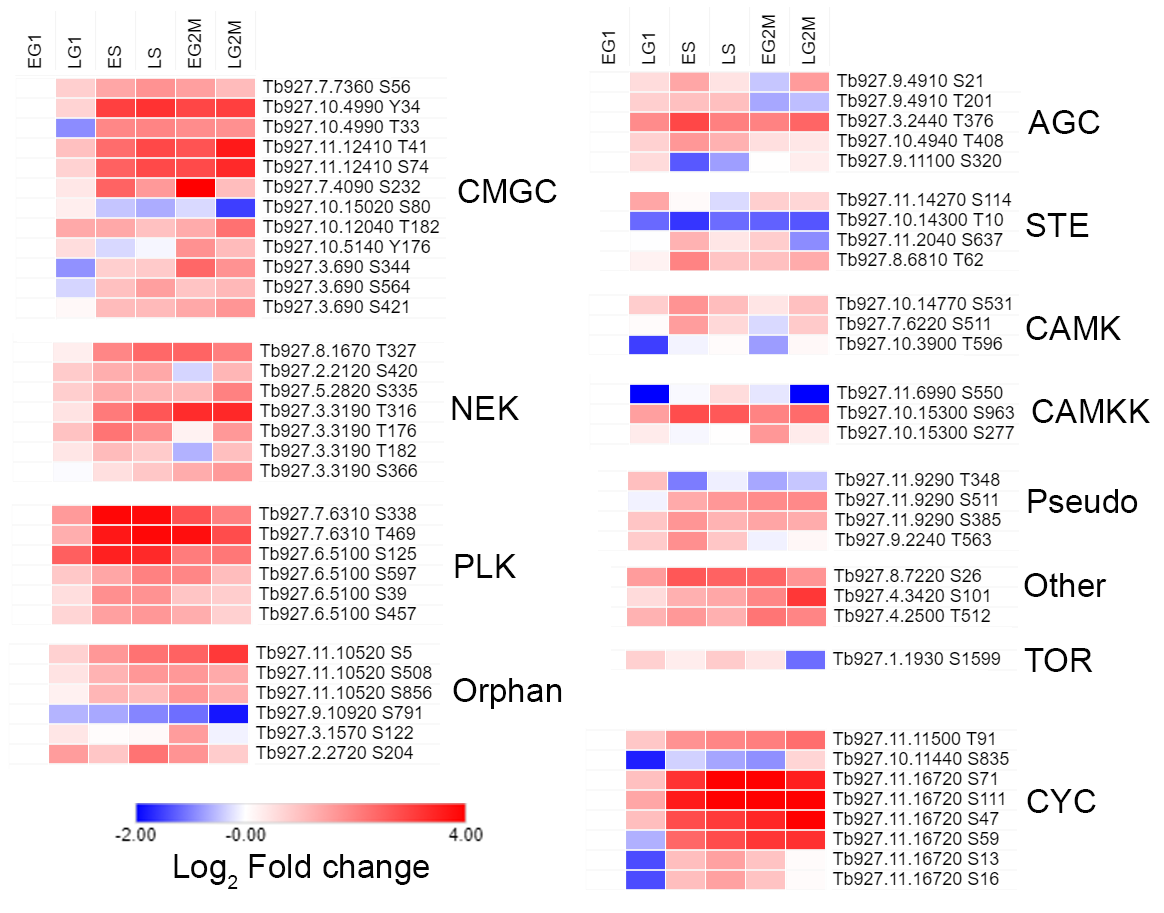

Supplement: S5 Fig — CCR protein kinase and cyclins rendered as a heat map of the log2 fold change relative to EG1, grouped by family. (TIF) [file ppat.1008129.s006.tif]

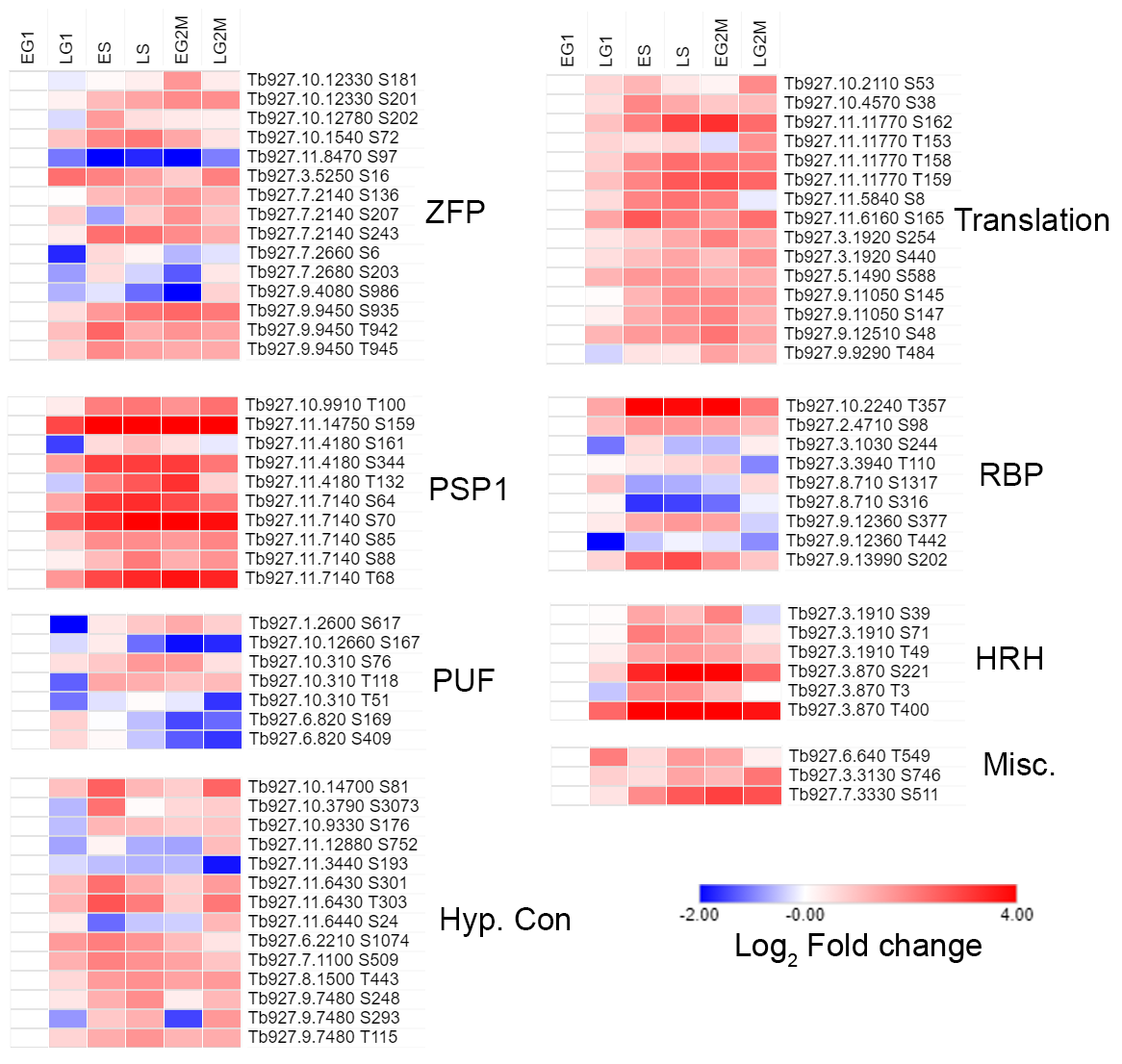

Supplement: S6 Fig — CCR Proteins containing recognisable RNA binding domains or identified from mRNA tethering screens and crosslinking proteomics [41–42] are rendered as a heat map of the log2 fold change relative to EG1, grouped by proteins features. ZFP–zinc finger proteins; Translation–eIF and associated proteins; PSP1 –PSP1 C-terminal domain; RBP–RNA binding motif; PUF—Pumilio/Fem-3 domain; HRH–Histone RNA hairpin; Hyp. Con–hypothetical conserved proteins; Misc.–miscellaneous. (TIF) [file ppat.1008129.s007.tif]

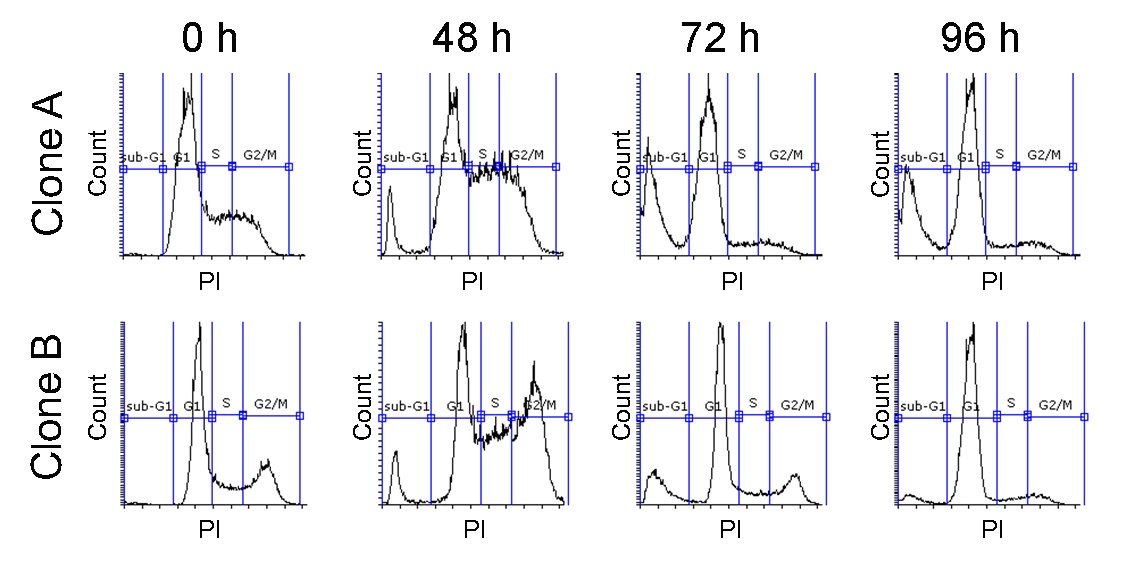

Supplement: S7 Fig — PI staining allows DNA content to be measured, demonstrating an accumulation of a sub-G1 population after 48 h of RNAi induction. (TIF) [file ppat.1008129.s008.tif]

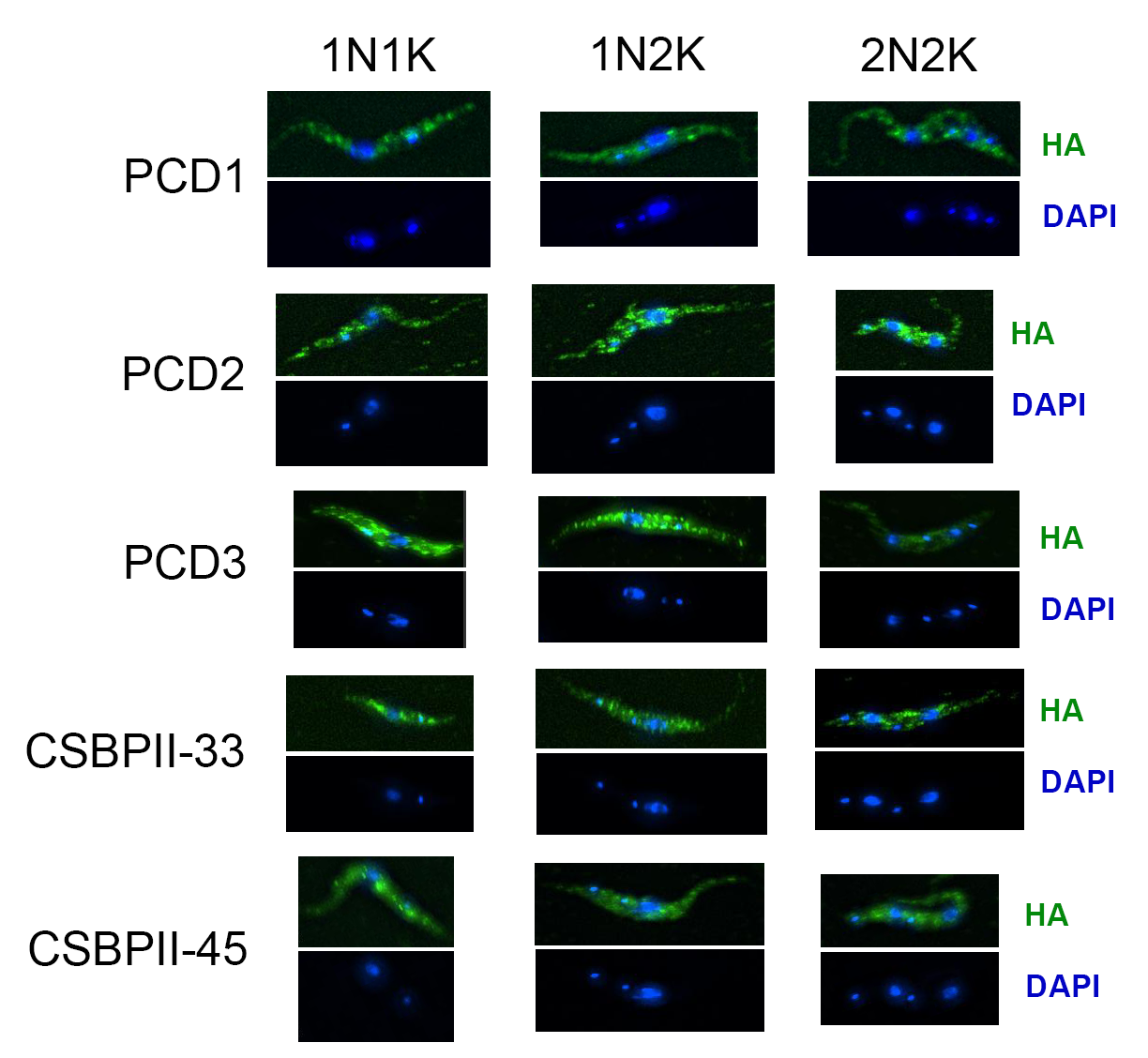

Supplement: S8 Fig — HA tagging endogenous tagging and immunofluorescence microscopy revealed the proteins have punctate localisation within the cytosol. No change in localisation occurred over the cell cycle, as judged by examining images with differing nucleus and kinetoplast counts. (TIF) [file ppat.1008129.s009.tif]

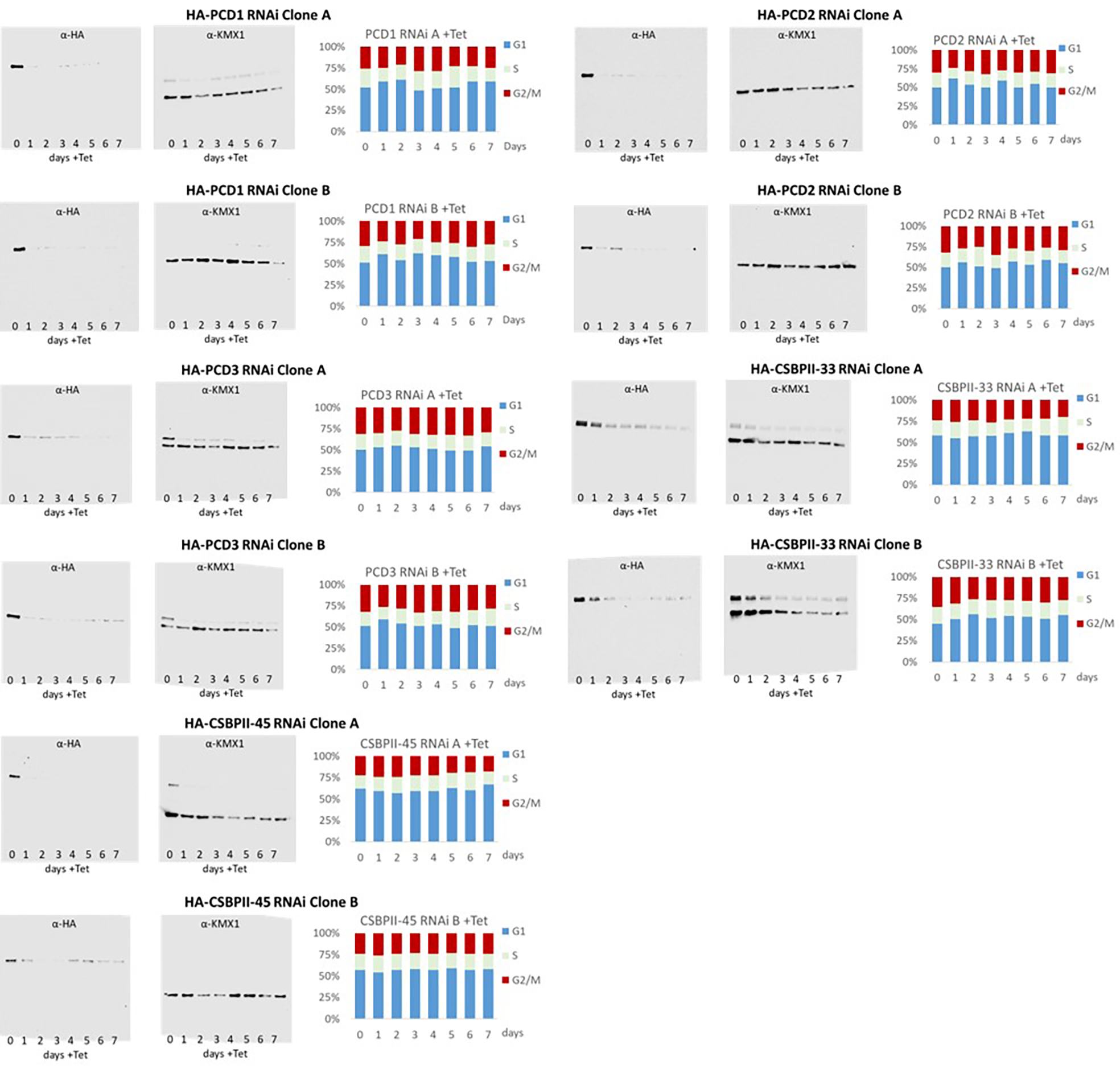

Supplement: S9 Fig — Aliquots of cells from the respective RNAi time course were subjected to Western blotting and flow cytometry. Western blots with anti-HA confirmed efficient knockdown of the HA-tagged proteins, with an anti-KMX-1 (tubulin) used a loading control. Flow cytometry of PI-stained cells revealed that the proportion of cells in different cell cycle time points was unchanged. (TIF) [file ppat.1008129.s010.tif]

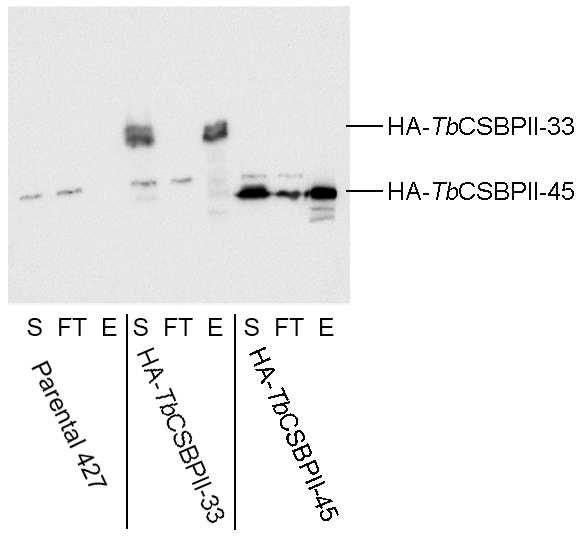

Supplement: S10 Fig — IP of HA-TbCSBPII-33, HA-TbCSBPII-45, and the parental 427 cells with anti-HA beads, subjected to anti-HA western blotting. HA-tagged proteins can be observed in the eluent. S–starting material; FT–flow through; E–eluent. (TIF) [file ppat.1008129.s011.tif]

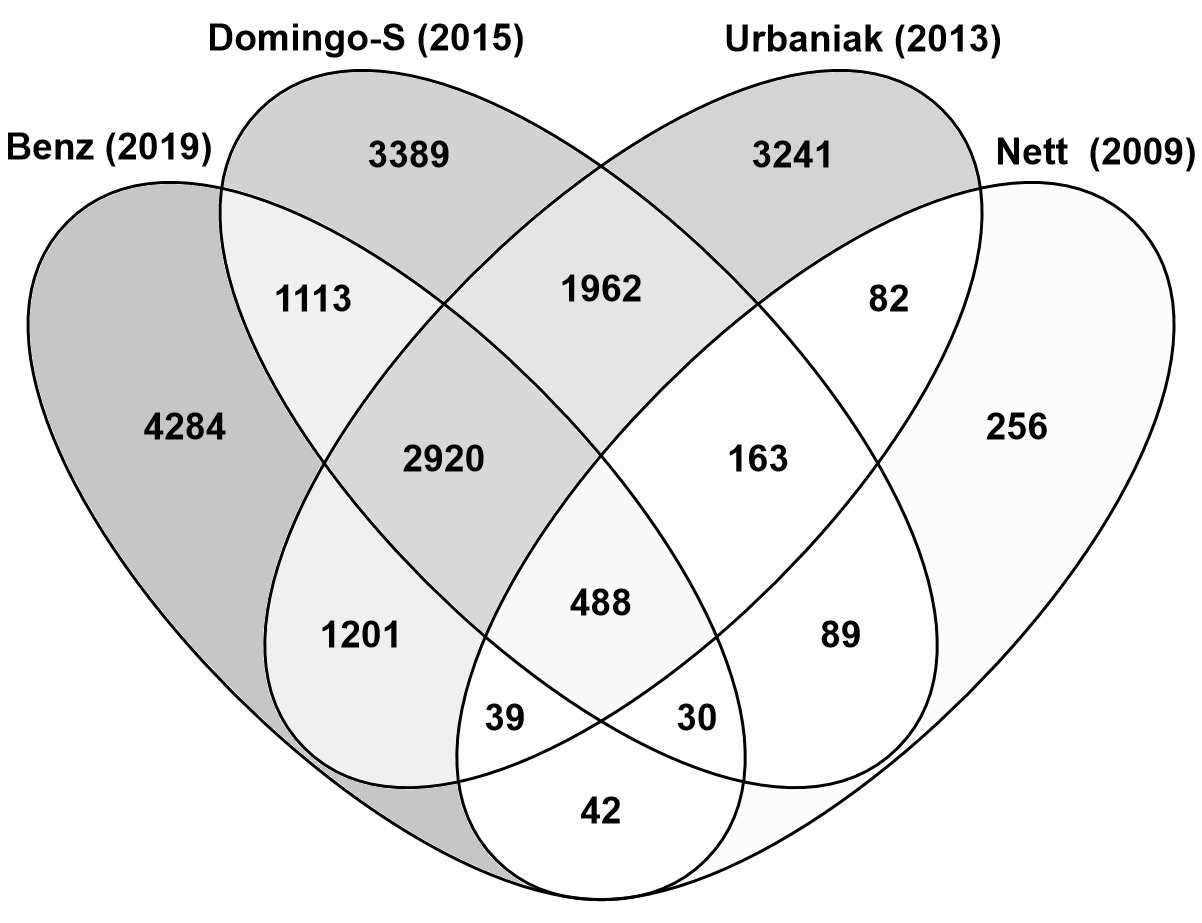

Supplement: S11 Fig — Benz (2019)– 10,119 phosphorylation site observed in synchronised Pcf cells (current study); Domingo-S (2015)– 10,159 phosphorylation sites observed in ex vivo Stumpy Bsf cells [52]; Urbaniak (2013)– 10,095 phosphorylation sites observed in Pcf and Bsf cells [18]; Nett (2009)– 1,190 sites observed in Bsf cells [53]. (TIF) [file ppat.1008129.s012.tif]

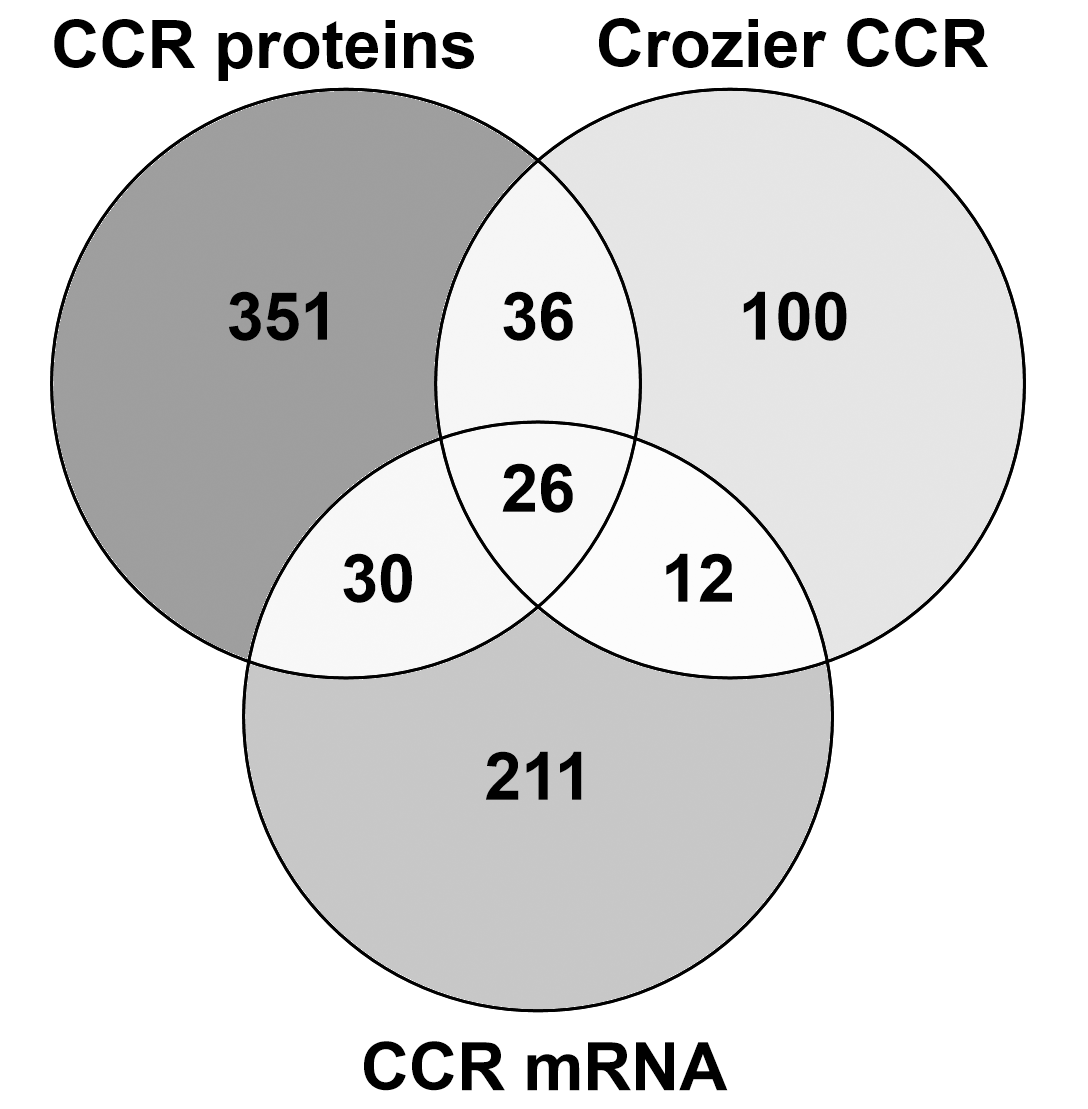

Supplement: S12 Fig — CCR proteins– 443 proteins identified in the present study; Crozier CCR– 174/384 CCR proteins reported by Crozier et al. [14] were quantified at all six time points; CCR mRNA– 279/528 CCR transcripts reported by Archer et al. [13] were quantified at all six time points. Shading represent percentage overlap. (TIF) [file ppat.1008129.s013.tif]
