# Supplementary material for: Organising the cell cycle in the absence of transcriptional control: Dynamic phosphorylation co-ordinates the Trypanosoma brucei cell cycle post-transcriptionally
Source: PLoS Pathog. 2019 Dec 12;15(12):e1008129. doi: 10.1371/journal.ppat.1008129 (PMC6907760; doi:10.1371/journal.ppat.1008129)
Supplement: S1 Table — (DOCX) [file ppat.1008129.s014.docx]

## **S1 Table**. Metadata for cell cycle proteomics samples.

| Sample Name | Sample origin^1^ | Proteomic Experiment | G1^2^ | S^2^ | G2/M^2^ |
| --- | --- | --- | --- | --- | --- |
| EG1-a | Direct, 18-20 ml/min | CC10-Heavy | 96 % | 3 % | 1 % |
| EG1-b | Direct, 18-20 ml/min | CC11-Heavy | 97 % | 2 % | 1 % |
| EG1-c | Direct, 18-20 ml/min | CC12-Light | 95 % | 2 % | 3 % |
| LG1-a | Direct, 22-24 ml/min | CC9-Heavy | 84 % | 14 % | 2 % |
| LG1-b | Direct, 22-24 ml/min | CC8-Heavy | 85 % | 12 % | 3 % |
| LG1-c | Direct, 22 ml/min | CC13-Light | 84 % | 11 % | 5 % |
| ES-a | Grown | CC14-Heavy | 45 % | 45 % | 10 % |
| ES-b | Grown | CC12-Heavy | 34 % | 45 % | 21 % |
| ES-c | Grown | CC6-Light | 40 % | 32 % | 28 % |
| LS-a | Grown | CC15-Heavy | 40 % | 48 % | 12 % |
| LS-b | Grown | CC14-Light | 22 % | 52 % | 26 % |
| LS-c | Grown | CC13-Heavy | 20 % | 46 % | 34 % |
| EG2M-a | Grown | CC9-Light | 16 % | 45 % | 39 % |
| EG2M-b | Grown | CC15-Light | 14 % | 47 % | 39 % |
| EG2M-c | Grown | CC8-Light | 19 % | 29 % | 52 % |
| LG2M-a | Grown | CC10-Light | 15 % | 25 % | 60 % |
| LG2M-b | Grown | CC11-Light | 24 % | 16 % | 60 % |
| LG2M-c | Direct, 25-35 ml/min | CC6-Heavy | 23 % | 12 % | 65 % |

^1^ Elutriation conditions; either directly eluted or eluted and placed back into culture

^2^ Cell cycle composition determined by flow cytometry of PI-stained sample.
