# Supplementary material for: Organising the cell cycle in the absence of transcriptional control: Dynamic phosphorylation co-ordinates the Trypanosoma brucei cell cycle post-transcriptionally
Source: PLoS Pathog. 2019 Dec 12;15(12):e1008129. doi: 10.1371/journal.ppat.1008129 (PMC6907760; doi:10.1371/journal.ppat.1008129)
Supplement: S7 Table — (DOCX) [file ppat.1008129.s020.docx]

**S7 Table**. PSP1-C terminal domain containing proteins present in *T. brucei*.

| Name | GeneDB ID | Proteome | Phosphorylation site | RBP^1^ |
| --- | --- | --- | --- | --- |
| **PCD1** | Tb927.11.14750 | LS | S159 | + |
| **PCD2** | Tb927.11.4180 | ES | S161, T132, S344 | + |
| **PCD3** | Tb927.10.8330 | ES | S81 |  |
| **PCD4** | Tb927.10.9910 | NC | T100 | + |
| ***Tb*CSBPII-33** | Tb927.11.7140 | NC | S64, T68, S70, S85, S88 | + |
| ***Tb*CSBPII-45** | Tb927.5.760 | NC | S85 |  |
| **PIE8** | Tb927.6.2850 | - | T144 |  |
| **PCD5** | Tb927.10.11630 | LG2M | - |  |
| **PCD6** | Tb927.8.3850 | - | - | + |
| **PCD7** | Tb927.3.5080 | - | - |  |
| **PCD8** | Tb927.9.9370 | - | - |  |

^1^ RNA binding proteins that interact directly with mRNA [41,42]. NC, Not changing; -, not observed; +, RBP.
