# Supplementary material for: Organising the cell cycle in the absence of transcriptional control: Dynamic phosphorylation co-ordinates the Trypanosoma brucei cell cycle post-transcriptionally
Source: PLoS Pathog. 2019 Dec 12;15(12):e1008129. doi: 10.1371/journal.ppat.1008129 (PMC6907760; doi:10.1371/journal.ppat.1008129)
Supplement: Attachment — Submitted filename: Response to reviewers comments.docx [file ppat.1008129.s021.docx]

Dr Michael D Urbaniak, FRSB

Biomedical & Life Sciences

Lancaster University

Bailrigg

Lancaster LA1 4YG

+44 (0)1524 593104

[m.urbaniak@lancaster.ac.uk](mailto:m.urbaniak@lancaster.ac.uk)

Dear Editors,

**R.E. Organising the cell cycle in the absence of transcriptional control: Dynamic phosphorylation co-ordinates the Trypanosoma brucei cell cycle post-transcriptionally (PPATHOGENS-D-19-01221)**

Please find attached a resubmission of the above research article for your consideration for publication in *PLoS Pathogens.* We would like to thank the reviewers and editors for their thorough reviews and helpful suggestions to improve the manuscript.

Below we have provided a detailed list of responses to the review comments and a description of the changes that we have made to the manuscript and supporting data. Attached are two versions of the manuscript, one with tracked changes, and the other a clean version. We can confirm that figures have been uploaded to PACE to ensure they meet PLoS requirements.

Best wishes,

Mick Urbaniak

***************

The referees find the manuscript to be interesting, of a very high standard and likely to be a valuable resource to the community. As you can see, the referees have raised several concerns. No further experimental work is required and no sections need to be removed, however, but it would be helpful to include some comparison with previously published proteomics datasets (including your own) and to address the issue of "thresholds". Reference to the data in Saldivia et al. (2019) bioRxiv 10.1101/616417 could provide the experimental validation for the role of phosphorylation in kinetochore function, which has been requested by one of the referees.

*Response:* We thank the Editors for sparing us the burden of performing extra experimental work. We can confirm that we have conducted a comparison with previously published proteomic data sets, addressed the issues of thresholds, and included the kinetochore BioRxiV reference – further details is provided in the response to referees below.

**Reviewer's Responses to Questions**

**Part I - Summary**

**Reviewer #1:** Benz and Urbaniak present quantitative proteomics and phosphoproteomics on procyclic Trypanosoma brucei cells synchronised by elutriation. They follow ~3600 proteins and ~6000 phosphosites through the full cell cycle, showing that phosphorylation changes are more numerous and of greater magnitude than protein level changes (or mRNA by comparison to previous work). Ordering the profiles according to time stage of maximum abundance, they describe the burst of phosphosites peaking during entry into S and M phases in particular and define 29 and 30 clusters of protein and phosphosite behaviours, respectively, some of which are associated with different gene ontologies. 2 phosphosite clusters with high-G2/M profiles are significantly enriched in likely CDK sites, while possible PLK sites are enriched in one of these and another which peaks during S phase.

The authors describe the detected protein/phosphosite changes occurring on particular sets of proteins: protein kinases, kinetochore proteins, RNA-binding proteins and components of the translation machinery. Based on some of these observations, they knockdown a protein with putative role in translation initiation (TbDED1.2) and show reduction in cells with DNA content expected for S and G2/M, and accumulation of cells with less than 2C content, concluding that might be due to this protein regulating transcripts in a phospho-dependent manner.

Finally, they describe a class of CCR proteins/phosphosites in trypanosomes, including previously described proteins associated with cycling transcripts, that are united by the presence of a PCP1 domain. Analysis of these proteins recapitulated profiles from the proteomics data, but produced no defect on depletion. Immunopurification of 2 of them (CSBPII-33 and -45) did not isolate the same complex as described in Crithidia, but showed a possible interaction with translation machinery.

How trypanosomes control the cell cycle is of clear scientific importance – especially given the divergent nature of many of the systems involved and that changes in mRNA (and even protein levels) probably don't contribute to the same degree as in other systems. The proteomics and phosphoproteomics data here appear to be of very high quality, and the extent and analysis of these data make a really substantial contribution to mapping and understanding this system. There are previous (non-cell cycle) phosphoproteomes published for trypanosomes [Nett et al. (2009) Mol Cell Proteomics, Urbaniak et al. (2013) J Proteome Res] and also a cell cycle proteome [Crozier et al. (2018) Mol Cell Proteomics], but these data are clearly distinct in scale and bringing together protein level changes with the first quantitation of phosphosite changes across the cell cycle makes this a dataset of considerable interest in an important model parasite that I can see gathering a lot of citations.

After the presentation of the proteomic data, however, I found the analyses of the specific protein sets increasingly descriptive and 'bitty'. The analysis of cell cycle regulation of kinases (and cyclins) was reasonably compelling, particularly as there was evidence of association of specific kinase motifs with specific cell cycle profiles (is there evidence of other motifs associated with unknown kinases that can be seen from these clusters?). The analysis of the kinetochore also seemed to add to the understanding of the system – although the analysis needs to incorporate recent data on KKT2 phosphorylation by CLK1/KKT10 deposited on bioRxiv (Saldivia et al. (2019) bioRxiv 10.1101/616417). However, data on RNA binding proteins and much of that on translation machinery was purely descriptive and should in my opinion be removed or moved to supplement. The data on TbDED1.2 added little to the manuscript and the conclusions are very speculative. The discovery of the association of PCP1 and cell cycle regulation is an interesting finding, although the lack of an observable defect on knockdown obviously leaves something of a mechanistic gap.

I realise that the attempt here is to add 'new biology' to avoid the proteomic data being dismissed as descriptive or too specialist. In my opinion, the quantitative proteomics here are of sufficient importance for understanding trypanosome (and leishmania) growth, that they cannot be dismissed in this way, and that some of the add-ons are actually detracting from these findings. I recommend that if these proteomics data are bolstered (see below) and some of the more speculative/descriptive elements later removed, this work is suitable for publication in PLoS Pathogens and likely to attract a good readership.

**Reviewer #2:** In the manuscript "Organising the cell cycle in the absence of transcriptional control: Dynamic phosphorylation co-ordinates the Trypanosoma brucei cell cycle post-transcriptionally", Benz & Urbaniak systematically study the contribution of protein phosphorylation to cell cycle regulation in African trypanosomes. Several studies that addressed changes in protein or transcription profiles during cell cycle progression exist already but to my knowledge, this is the first approach to evaluate the function of dynamic phosphorylation during this process. Synchronisation of the cells by elutriation is a well-established and suitable method for this purpose. The authors identified more than 900 cell cycle-regulated phosphorylation sites, some of them in proteins that have not been associated with cell cycle control, yet. Although this study is mainly descriptive, this set of data is without doubt a valuable resource for the community.

The manuscript is well written and concise. The design of the study is in general well thought though and performed with high experimental quality and adequate sample size. Since I am not a MS expert, I cannot comment on technical details or suitability of bioinformatic methods.

There is nothing wrong with the descriptive part of the study (clustering of the phosphorylation site, cell cycle regulation of kinases and dynamic phosphorylation of specific protein groups). However, an experiment to confirm that any the novel CCR phosphorylation sites are indeed involved in cell cycle control would improve the manuscript substantially (see below).

**Reviewer #3:** The manuscript describes:

1. An analysis of changes in protein abundance over a cell cycle in elutriation synchronised cells.

2. The identification of protein phosphorylation sites that vary over the cell cycle

3. Some tests of the observations (or hypotheses drawn from the measurements) using reverse genetics.

The work is meticulous and the measurements described with clarity. The validity of the measurements are discussed. I enjoyed reading the manuscript and it an exemplary mass spec analysis of an very interesting biological phenomenon.

________________________________________

**Part II – Major Issues: Key Experiments Required for Acceptance**
Please use this section to detail the key new experiments or modifications of existing experiments that should be absolutely required to validate study conclusions.

**Reviewer #1:** The thresholds for being ‘cell cycle regulated’ appear arbitrary (3-fold for phosphosites and 1.5-fold for protein). To a certain extent these will always be the case, but a substantial portion of the conclusions in the manuscript are based on comparisons of either protein or phosphosites that are ‘CCR’ (either overall numbers or specific proteins) – or comparison to other sets which use different thresholds (e.g. Crozier et al. (2018) Mol Cell Proteomics wherein a 1.3-fold threshold is used). As such, some rationale should be provided as to why these thresholds have been used. Or at least some exploration of how the conclusions might be affected by implementation of alternative thresholds. It would also be very helpful to see some representation of the distribution of the data with respect to the threshold somewhere in the manuscript rather than only presenting already filtered data.

The proteomics data add to/supersede three related (phospho)proteomic datasets [Nett et al. (2009) Mol Cell Proteomics, Urbaniak et al. (2013) J Proteome Res, Crozier et al. (2018) Mol Cell Proteomics]. Some comparison to Crozier et al. is made in the discussion, and it is not necessarily easy to make direct comparisons due to difference in methodology, but it would be extremely useful to the reader to see more information on how these data relate to previous. Which of the phosphosites seen in the non-cell cycle data are also seen in these data? The number of observed sites has increased, but what is the overlap? Is there any link between CCR phosphosites and those changing between lifecycle stage? As mentioned, there is little overlap between the CCR protein set here and in Crozier et al., but how much of this might be thresholding effects? Is their correlation in CCR? It is not necessary to address all these question, but some consideration would greatly aid the reader in navigating between the sets.

*Response:* We attempt to address all of the many interrelated points raised by the reviewer below:

*Rational for threshold level*

We did not employ a probability-based statistical threshold to define cell cycle regulation as the temporal data are not normally distributed, as revealed by plotting a histogram of Log_2_ FC_Max_, but defined species greater than twice the median value as cell cycle regulated. The Log_2_ FC_Max_ values for all proteins and phosphorylation sites are already included in Table S2 and Table S3, and we have added the histograms showing the distribution of Log_2_ FC_Max_ as an additional figure (Fig S1). We also note that the threshold chosen by Crozier et al of FC_Max_ 1.3 appears to be arbitrary.

We have added the following text to the Supplementary methods section ‘Selection of cell cycle regulated proteins and phosphorylation sites’:

“A histogram of Log_2_ FC_Max_ demonstrated a non-normal distribution of data, with a peak centred on the median value that was skewed toward higher values (Fig S1). We defined any protein with an FC_Max_ ≥ 1.5-fold or phosphorylation site with FC_Max_ ≥ 3-fold as cell cycle regulated (CCR), threshold values that represent approximately twice the median FC_Max_ (2 × Median protein FC_Max_ = 1.44; 2 × Median phosphorylation site FC_Max_ = 2.99).

Fig S1. Histogram of Log_2_ FC_Max_ demonstrating non-normal data distribution. A Protein FC_Max_; B Phosphorylation site FC_Max_. Blue – Non –regulated, Red – Cell cycle regulated.”

*Implementation of alternative thresholds*

Crozier et al. identify 384 CCR proteins with fold change (FC) >1.3; we observe 255 (66%) of these in our proteomic data, 174 (45%) of which we can quantify at all six time points. Using our current threshold of FC >1.5 we identify 443 CCR proteins of which 62 (14.7%) are in common with Crozier et al. with a modest Pearson correlation of 0.539 between the fold changes observed. To illustrate the effect of implementing an alternative threshold, if we relaxed our threshold to FC >1.3 there are 1048 CCR proteins of which 95 (9.6%, Pearson correlation 0.546) are in common with Crozier et al. Thus lowering the threshold increases the number in common without significantly changing the correlation, but results in a decrease in the percentage overlap between the two CCR protein sets. Overall, we believe it is not possible to disentangle the overlapping effects of “differences in methodology, criteria used to select CCR proteins, and the stochastic nature of data dependent mass spectrometry” as we state in L532-534.

The Pearson correlation has been added to the discussion (underlined):

“We only observe 179 of these CCR proteins at all six time points and only classify 62 of them as CCR (>1.5-fold change, Pearson correlation 0.546)…”

*Comparison to published phosphoproteomic data sets:*

We have compared the current data to previous global studies, but do not find any correlation between CCR phosphosites and those changing between lifecycle stage.

The following paragraph has been added to the discussion:

“ Our analysis of cell cycle synchronised Pcf cells identifies a total of 10,119 phosphorylation sites, of which 4,284 have not previously been reported including 408 CCR sites. The total number of phosphorylation sites observed is comparable to our previous studies where 10,095 phosphorylation sites were identified from asynchronous Pcf and bloodstream form (Bsf) cells (18) and 10,159 phosphorylation sites identified from *ex vivo* stumpy Bsf cells that identified 1,204 phosphorylation sites (51), and is an order of magnitude greater than the first global study Bsf *T. brucei* (52). Each study is able to identify unique sites (Fig S11), consistent with the difference in sample origin, processing and data analysis, suggesting that many phosphorylation site remain to be identified. The relatively similar overall total number of sites identified in our phosphoproteomic studies may reflect a limit to the number of sites it is possible to identify in a single study using the current methodology.”

Figure S10. Venn diagram of the overlap of global phosphoproteomic studies. Benz (2019) – 10,119 phosphorylation site observed in synchronised Pcf cells (current study); Domingo-S (2015) – 10,159 phosphorylation sites observed in *ex vivo* Stumpy Bsf cells (19); Urbaniak (2013) – 10,095 phosphorylation sites observed in Pcf and Bsf cells (2); Nett (2009) – 1,204 sites observed in Bsf cells (20).”

The profiles of phosphosite/proteins are really useful in understanding the cell cycle and being able to conceptualise them as archetypes is in my opinion a substantial contribution – as exemplified by the ability to see associate specific phosphosite motifs to particular archetypes (although this is not explored further). A small number of these profile clusters are shown in Fig.4, but I recommend that they are all liberated from the supplement and brought to the main manuscript. In addition, there is clearly information in the relationships between the clusters (for example in the relative similarity of 855 and 874, and their dissimilarity to 862/884) and between protein-level and phosphosite clusters. It would be really useful to see the heirarchical structure of the similarities between the clusters, and especially in the similarities between phosphosite behaviours and protein profiles. How do the clusters relate to the ordering based on peak time seen in Fig 2?

*Response:* We are pleased that the Referee finds the hierarchical clustering of value. We have replaced Fig 4 with a new composite figure that graphically display the hierarchical relationship between the phosphorylation site clusters by rendering the hierarchy as a tree and individual profiles as a heatmap, as well as showing the GO term enrichment and profile of each cluster. The corresponding tree and heatmap for protein clusters is an additional figure in the supplementary data (Fig S2), the profiles in Fig S3 and GO term enrichment in a modified Fig S4 . The clusters have also been renamed as requested (see below).

The relationship between peak time and clustering is complex; peak time is time in the cell cycle at which the CCR protein/phosphorylation site abundances reaches a maximum, whilst the clustering takes into account the complete profile across the cell cycle. Both peak time and cluster identifiers are given in Table S2 and S3.

Additional/modified figures:

“Fig 4. Hierarchical clustering of cell cycle regulated phosphorylation sites. A. Unrestrained hierarchical clustering was performed using Euclidean distance of the complete linkage, generating 30 clusters (Phos-01 to Phos-30). The relationship between the clusters is rendered as a tree, and the profiles are rendered as a heat map. B. Gene Ontology (GO) enrichment analysis of clusters Phos-01 – Phos-06 (red bars), containing >50% of the CCR phosphorylation sites, identifies GO terms related to cell cycle and mRNA. C. Individual phosphorylation sites profiles by cluster; number of cluster members is given in brackets, and the profiles are coloured by the Euclidean distance from the centre (mean profile).”

“Fig S2. Hierarchical clustering of cell cycle regulated proteins. The protein ratios were Z-transformed and unrestrained hierarchical clustering performed using Euclidean distance of the complete linkage, with 29 clusters (Prot-01 to Prot-30) defined using a minimum distance threshold of < 2.5. The relationship between the clusters is rendered as a tree, and the Z-transformed phosphorylation site profiles are represented as a heat map. Image prepared in Perseus (10).”

“Fig S4. Comparison of Gene Ontology enrichment in cell cycle regulated protein clusters. Gene Ontology (GO) enrichment analysis using GO Slim ontology with *P* < 0.05 was performed on the largest clusters representing >50% of the CCR proteins (red bars), and the occurrence of GO terms related to cell cycle tabulated.”

In my opinion, the sections “Dynamic phosphorylation of RNA binding proteins” and “Dynamic phosphorylation of translation machinery” should be removed or, if not removed, substantially augmented to produce more tangible mechanistic insight. If the data are kept, some measure of significance for the observed changes must be included.

*Response:* We are disappointed that the Referee cannot see the value of these sections, as we feel that they do offer mechanistic insights. We have not removed these sections, as directed by the Editors, but have included a measurement of the significance of the observed changes using Fisher’s exact test of categorical enrichment with a Benjamini-Hochberg correction.

The following text has been added:

“The CCR phosphorylation sites are significantly enriched for RBPs (Benjamini-Hochberg FDR 9.2 × 10^-3^) and include 79 CCR phosphorylation sites (average FC_Max_ 5-fold) on 51 RNA binding proteins (RBPs). Likewise, CCR regulated proteins are significantly enriched for RBPs (Benjamini-Hochberg FDR 7.8 × 10^-3^) and include 13 RBPs are CCR at the protein level (Supplemental Figure S5).”

“Amongst the RBPs with CCR phosphorylation sites there is a significant enrichment of the GO term “translational initiation factor activity” (Benjamini-Hochberg FDR 2.7 × 10^-3^).”

Given that classification of DNA content into G1, S, G2/M by flow cytometry is highly dependent on the sample preparation, gating, etc. histograms of the raw data should also be included in supplement. Surely the distinction between 'failure of replication or stalled mitosis' can be made from the flow cytometry data?

*Response:* We have added an additional supplementary figure (Fig S7) of the histograms of the raw flow cytometry data and regions used to define the cell cycle position. We state that “cells are failing to correctly complete G2/M, potentially due to failure of replication or stalled mitosis, but subsequently proceed through cytokinesis to produce cells with insufficient nuclear material.” We observed the accumulation of cells with insufficient nuclear material by IF and FACS, but do not follow the fate of individual cells with either technique and so cannot conclude whether these cells arise due to failed replication followed by mitosis & cytokinesis, or by correct replication and stalled mitosis followed by cytokinesis.

The following figure has been added to the supplementary data:

“Figure S7 – Flow cytometry analysis DED1.2 RNAi time course. PI staining allows DNA content to be measured, demonstrating an accumulation of a sub-G1 population after 48 h of RNAi induction.”

Non-essentiality cannot be inferred from knockdown [line 471/2] as it is unclear how much protein would be required to fulfil any (unknown) function.

*Response:* We have demonstrated that knockdown of the five PSP1-C domain containing proteins using inducible RNAi ablates the tagged proteins to levels undetectable by western blotting (Fig 9 & S7), yet do not observe any significant growth defect or cell cycle defect for 7 days (~21 cell cycles). Therefore, we feel it is justified to infer that the individual proteins are not essential for successful completion of the cell cycle.

CSBPII-33 shows clear co-IP with PCD4, but other than that it is unclear which interactions are genuine and which noise. Interaction with ribosomal proteins is consistent with the proposed role in translation, but these are also very high abundance proteins that commonly contaminate IP. Since neither the anticipated interaction between CSBPII-33 and -45 or clear binding to PABP2 is seen, more data will be needed to either discriminate more clear signal/noise or show that tagging has not disrupted function (an alternative tag at the C-terminus?).

*Response:* We disagree that the ribosomal proteins are contaminants. Crucially, our IP experiments use SILAC labelling to distinguish between high abundance low affinity binders and genuine interactions; contaminants will bind equally to the beads and have a SILAC ratio close to unity (i.e. H/L = 1), whereas genuine interactions will yield a higher ratio (i.e. H/L >1), with label swaps in biological replicates used to eliminate systematic bias. The high level of enrichment of the target protein confirm that efficient IP has occurred, and the high SILAC ratios of the ribosomal proteins are consistent with a specific interaction. Furthermore, a different set of ribosomal proteins are enriched with IP of each of the two HA-tagged proteins, and the different sets are reproducibly isolated in each biological replicate.

We agree that we cannot eliminate the possibility that the introduction of the small HA tag has disrupted the function of the tagged protein. However, it is unlikely that such disruption would only affect the interaction of *Tb*CSBPII-33 with *Tb*CSBPII-45 and yet not affect the interaction of *Tb*CSBPII-33 with PCD4. We did not tag at the C-terminus in order to maintain the native 3’UTR that are often important for regulation in *T. brucei*.

The following text has been added (underlined):

“Whilst we cannot eliminate the possibility that the introduction of the HA tag has disrupted the function of the tagged proteins, the co-immunoprecipitation of *Tb*CSBPII-33 and PCD4 is consistent with formation of a *Tb*CSBPII-33 – PCD4 complex that is likely to include mRNA, and that interacts with the translation machinery in the ribosome as well as other RBPs including PABP2.”

**Reviewer #2:** line123: the authors claim that cells "maintain synchrony into subsequent cell cycles". This is a little misleading. To my knowledge, cells lose synchrony quickly within a few cell cycles. The authors should show the data or state this more precisely.

*Response:* We have demonstrated that synchronicity is maintained into the third cell cycle following our elutriation protocol in Benz *et al* (Ref 19). For clarity the text has been altered (underlined)

“…maintain synchrony into three subsequent cell cycles (19)"

line 287 As mentioned above, ONE experiment to confirm that the identified CCR phosphorylation sites are indeed involved in cell cycle control would improve the manuscript substantially. For example, would over expression of MAPK6 or RCK mutants without their CCR phosphorylation sites show a dominant negative (cytokinesis) phenotype?

*Response:* We agree that the experimental demonstration that the identified CCR phosphorylation sites are directly involved in cell cycle control would improve the manuscript. However, this is non-trivial to achieve and represents a substantial piece of additional work, and we disagree that this “should be absolutely required” to validate the work and allow publication of the dataset.

We have not carried out any additional experimental work, as directed by the Editors.

line 302: A similar experiments is possible with the identified kinetochore protein phosphorylation sites, which may play a role in kinetochore assembly (or maybe not).

*Response:* See response above.

We have incorporate recent experimental data confirming a role for KKT2 S508 phosphorylation in kinetochore assembly and function by Saldivia et al. (2019) bioRxiv 10.1101/616417.

The following text has been added:

“Recently, Saldivia *et al* have shown that the phosphorylation of KKT2 S508 is essential for KKT2 function and kinetochore assembly, and that KKT2 S508 is phosphorylated by CLK1/KKT10 (40).”

**Reviewer #3:** There are no major issues

*Response:* Thank you, we agree.

**Part III – Minor Issues: Editorial and Data Presentation Modifications**
Please use this section for editorial suggestions as well as relatively minor modifications of existing data that would enhance clarity.

**Reviewer #1:** The naming of the clusters is unhelpful - e.g. 30 clusters for phosphosite profiles with ids in the range 735-886. I assume these are in some way linked to something “under the hood” of the analysis, but it doesn't aid in comprehension. Could the authors consider changing? It would be really useful to have some obvious distinction in the naming between clusters at the protein level and those at the phosphosite.

*Response:* We have renamed the phosphorylation site clusters to Phos-01 to Phos-30 in order of descending cluster size, and the protein clusters to Prot-01 to Prot-29, and updated the figures, text and data files accordingly.

Given the apparent usefulness of the clusters, why are they only used in the section on protein kinases?

*Response:* The hierarchical clustering is particularly useful in the protein kinase section as there is a correlation between kinase activity and the enrichment of phosphorylation on canonical kinase sequence recognition motifs. The clusters are also used in the GO term enrichment analysis and mentioned when appropriate in other sections.

To which clusters do the kinetochore kinases belong?

*Response:*

Phosphorylation sites on KKT10 belong to Cluster 885 and Cluster 874, those on KKT2 belong to Cluster 885 and Cluster 874, and those on KKT3 Cluster 862. KKT10 and KKT19 protein abundance both belong to Cluster 392. The data is reported in Table S4 (phosphorylation sites) and Table S5 (protein abundance) which can be sorted on ‘Kinetochore’ column.

Do most kinetochore proteins belong to the same cluster?

*Response:* The 22 cell cycle regulated phosphorylation sites on kinetochore proteins belong to 8 different phosphorylation site clusters (L292 – 296), and the 5 kinetochore proteins cell cycle regulated at the protein abundance level occur in two clusters. The data is reported in Table S4 (phosphorylation sites) and Table S5 (protein abundance) which can be sorted on ‘Kinetochore’ column.

What are the 5 sites with >100-fold change excluded from Fig 2 and where in the cycle do they fall?

*Response:* The 5 sites with >100-fold abundance are from four hypothetical proteins. Details of their maximum fold change, peak time and cluster are reported in Table S4.

Currently, the PRIDE ID does not resolve to any data. I’m assuming this is only that the data are embargoed, but would need resolving before publication.

*Response:* The PRIDE data is currently not public, so a reviewer ID and password were provided in the cover letter that appears not to have been provided to reviewers. The data set will become publically available immediately upon publication.

There is a weird distinction between “cell cycle” (e.g. RNA binding) and “unrelated” (e.g. microtubule organizing center) in the GO terms in Fig.4. How is this distinction being made?

*Response:* The distinction between GO terms related to the cell cycle and those unrelated was made using our own biological judgement, but all significantly enriched (*P* < 0.05) terms were shown in the figure. We did not include “microtubule organising center” as we felt this would encompass both cell cycle related and structural functions.

We did not intend to claim that GO terms related to RNA were terms that should be considered as cell cycle regulated, but were instead highlighting the presence of GO terms relating to RNA in the same clusters where GO terms relating to the cell cycle (see L208 - 210). As Fig 4 has been altered, no changes have been made.

**Reviewer #2:** Fig1B I assume that the percentage of cells in a specific cell cycle is based on the gates set in Fig1A. Has that been confirmed by nuclei/kinetoplast configuration?

*Response:* The flow cytometry profiles (Fig 1A) were used to determine the percentage of cells in G1, S and G2/M in the nuclear cell cycle (Fig 1B), and the composition of each biological replicate is given in Table S1. We did not conduct nucleus and kinetoplast counts for these samples, but have established in Benz *et al.* (Ref 19) that the results of analysis of DNA content by flow cytometry or nucleus and kinetoplast counting are consistent.

line 173/174: It is an over-interpretation to claim that changes in phosphorylation are more important that changes in in protein abundance (based on on the profiles) without functional data

*Response:* We have altered the text (underlined) to clarify the intended point.

“..suggesting that changes in protein phosphorylation are more closely correlated with progression through the cell cycle than changes in protein abundance.”

**Reviewer #3:** Line 121: ‘high quality population’ - what does this mean? Give a percentage of cells that fitted within the defined parameters for G1

*Response:* The quality of a population of synchronised cells may be judged by the uniformity of size and DNA content, and ability to maintain synchronicity into subsequent cell cycles and was established in detail in Ref 19.

The percentage of G1 cells is now given:

“(95-97% G1)”

Line 241: a few words to explain T loop

*Response:* The text has been altered (underlined) to include a brief explanation.

“No phosphorylation of CRK3 occurred at the T-loop threonine residue, which typically is required to increases CDK activity, consistent with the observation that T-loop phosphorylation is not required for *Leishmania* CRK3 kinase activity (30)”

Line 248: how might the lack of the 3 phosphorylation sites contribute to the metaphase arrest in caused by the destruction box deleted cyclin

*Response:* The truncation of CYC6 results in the removal of CCR phosphorylation sites S13, S16 and S47 (FC_Max_ ≥ 7-fold) in addition to the putative destruction box. The removal of six negative charges from the C-terminal domain that are cell cycle regulated is likely to disrupt normal regulation of CYC6.

The text has been amended (underlined):

“Expression of a truncated CYC6 protein fusion (GFP-NLS-CYC6^Δ1-57^) to remove a putative destruction box (residues 54-57) arrests the nucleus in a metaphase-like state but does not prevent cytokinesis (31), and the lack of the S13, S16 and S47 CCR phosphorylation sites may contribute to this effect through the loss of CCR negative charge that results.

Line 262: reference needed

*Response:* This sentence is a conclusion drawn from our own work.

The text has been altered for clarity (underlined):

“However, the absence of CRK3 T-loop phosphorylation and the temporal correlation of canonical CDK activity with CRK3 phosphorylation on pT33 and pY34 suggest that alternative mechanisms of control may operate.”

Line 403: delete ‘are’

*Response:* Deleted

Line 452: why is a punctuate pattern in the cytoplasm consistent with mRNA binding?

*Response:* The majority of mRNA binding proteins are expected to be found in the cytoplasm, and previous observation of well characterised RBPs suggest a punctate cytoplasmic pattern is common i.e PUF9, RBP6, PABP2

Line 550: the discussion of the differences between datasets from different labs is very useful. These dirty little secrets need more open discussion. Perhaps the authors could give the overlap between the phosphorylation sites detected here and their earlier paper to provide information on contributors to variation.

*Response:* The overlap between the current data and previous phosphoproteomic data sets is now provided in the discussion and Fig S11 (see above)

Very minor points

The authors often use ‘results’ when they mean measurements or observations and ‘Our data show’ when they mean their interpretation of the data

Finally, and to be very pedantic, data is plural.

*Response:* The referee makes a valid point regarding our choice of language and we have made appropriate modifications in numerous places in the text.
